# Ubiquitin Ligases of the N-End Rule Pathway: Assessment of Mutations in *UBR1* That Cause the Johanson-Blizzard Syndrome

**DOI:** 10.1371/journal.pone.0024925

**Published:** 2011-09-13

**Authors:** Cheol-Sang Hwang, Maja Sukalo, Olga Batygin, Marie-Claude Addor, Han Brunner, Antonio Perez Aytes, Julia Mayerle, Hyun Kyu Song, Alexander Varshavsky, Martin Zenker

**Affiliations:** 1 Division of Biology, California Institute of Technology, Pasadena, California, United States of America; 2 Institute of Human Genetics, University Hospital, Magdeburg, Germany; 3 Service de Génétique Médicale, CHUV, Lausanne, Switzerland; 4 Department of Human Genetics, University Medical Center Nijmegen, Nijmegen, The Netherlands; 5 Dismorfologia y Genetica Reproductiva, Grupo de Investigacion en Perinatologia, Instituto de Investigacion Sanitari, Fundacion Hospital La Fe, Valencia, Spain; 6 Department of Gastroenterology and Nutrition, University Hospital, Greifswald, Germany; 7 School of Life Sciences and Biotechnology, Korea University, Seoul, South Korea; 8 Institute of Human Genetics, University Hospital Erlangen, University of Erlangen-Nuremberg, Erlangen, Germany; University of Tuebingen, Germany

## Abstract

**Background:**

Johanson-Blizzard syndrome (JBS; OMIM 243800) is an autosomal recessive disorder that includes congenital exocrine pancreatic insufficiency, facial dysmorphism with the characteristic nasal wing hypoplasia, multiple malformations, and frequent mental retardation. Our previous work has shown that JBS is caused by mutations in human *UBR1*, which encodes one of the E3 ubiquitin ligases of the N-end rule pathway. The N-end rule relates the regulation of the *in vivo* half-life of a protein to the identity of its N-terminal residue. One class of degradation signals (degrons) recognized by UBR1 are destabilizing N-terminal residues of protein substrates.

**Methodology/Principal Findings:**

Most JBS-causing alterations of UBR1 are nonsense, frameshift or splice-site mutations that abolish UBR1 activity. We report here missense mutations of human *UBR1* in patients with milder variants of JBS. These single-residue changes, including a previously reported missense mutation, involve positions in the RING-H2 and UBR domains of UBR1 that are conserved among eukaryotes. Taking advantage of this conservation, we constructed alleles of the yeast *Saccharomyces cerevisiae UBR1* that were counterparts of missense JBS-*UBR1* alleles. Among these yeast Ubr1 mutants, one of them (H160R) was inactive in yeast-based activity assays, the other one (Q1224E) had a detectable but weak activity, and the third one (V146L) exhibited a decreased but significant activity, in agreement with manifestations of JBS in the corresponding JBS patients.

**Conclusions/Significance:**

These results, made possible by modeling defects of a human ubiquitin ligase in its yeast counterpart, verified and confirmed the relevance of specific missense *UBR1* alleles to JBS, and suggested that a residual activity of a missense allele is causally associated with milder variants of JBS.

## Introduction

Johanson-Blizzard syndrome (JBS; OMIM 243800) is a rare autosomal recessive genetic disease of multiple congenital malformations. A combination of nasal wing aplasia and exocrine pancreatic insufficiency is particularly characteristic of JBS. Other commonly encountered JBS features include short stature, oligodontia, deafness, scalp defects, hypothyroidism, imperforate anus, genitourinary malformations, and frequent mental retardation [1]–[5]. Our previous work [6] and subsequent studies [7]–[9] have shown that JBS results from homozygous or compound heterozygous mutations in human *UBR1*, which encodes one of the E3 ubiquitin (Ub) ligases of the N-end rule pathway [10], [11]. We also found an exocrine pancreatic insufficiency in *Ubr1^−/−^* mice that lacked Ubr1, a phenotype similar to but less severe than the pancreatic phenotype of JBS patients that apparently lack active UBR1 [6].

**Figure 1:**
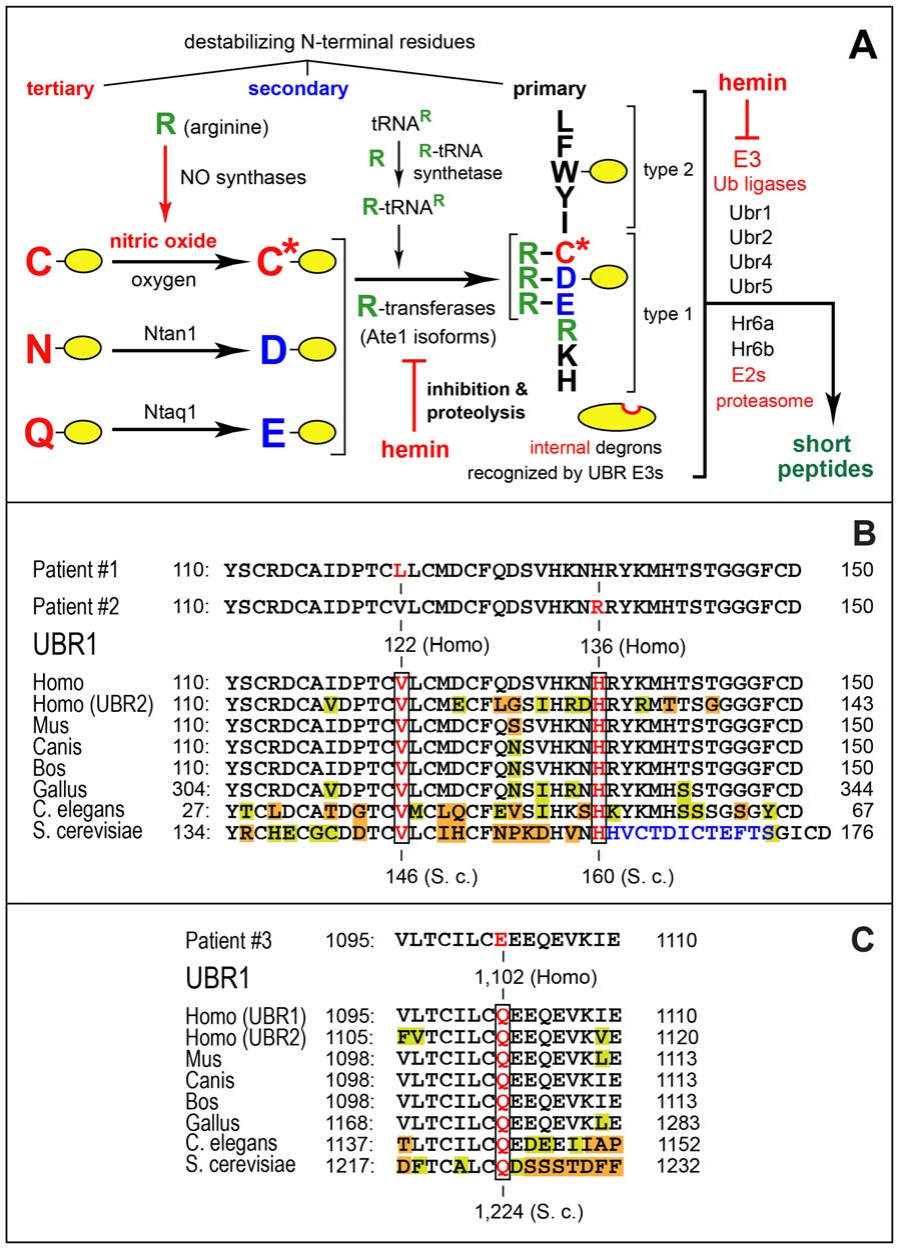
The mammalian Arg/N-end rule pathway and missense mutations in human *UBR1* that underlie specific cases of the Johanson-Blizzard syndrome (JBS). (A) The mammalian N-end rule pathway. N-terminal residues are indicated by single-letter abbreviations for amino acids. Yellow ovals denote the rest of a protein substrate. ‘Primary’, ‘secondary’ and ‘tertiary’ denote mechanistically distinct subsets of destabilizing N-terminal residues (see Introduction). C* denotes oxidized Cys, either Cys-sulfinate or Cys-sulfonate. MetAPs, Met-aminopeptidases. (B) Single-residue mutations in the UBR1 proteins of JBS patients #1 and #2. The positions of mutant residues are indicated both for the original mutations in human UBR1 and for their mimics in *S. cerevisiae*. (C) Same as in B but the mutation in UBR1 of patient #3 (see Results).

The N-end rule relates the regulation of the *in vivo* half-life of an intracellular protein to the identity of its N-terminal residue [12]–[20]. In eukaryotes, the N-end rule pathway is a part of the Ub system, which mediates protein turnover through the conjugation of Ub, a 76-residue protein, to proteins that contain specific degradation signals (degrons), thereby marking these proteins for degradation by the 26S proteasome [16], [17], [21]–[27]. N-terminal degrons recognized by the N-end rule pathway are called N-degrons. The main determinant of an N-degron is a destabilizing N-terminal residue of a protein (Fig. 1A). Recognition components of the N-end rule pathway are called N-recognins. In eukaryotes, N-recognins are E3 Ub ligases that bind to specific N-degrons [11], [15]–[17], [28]–[34].

The N-end rule pathway consists of two branches, the Ac/N-end rule and the Arg/N-end rule pathways. The Ac/N-end rule pathway targets proteins containing N^α^-terminally acetylated (Nt-acetylated) residues [17], [32]. It involves the cotranslational Nt-acetylation of nascent proteins [35]–[43] whose N-termini bear either Met or the small uncharged residues Ala, Val, Ser, Thr or Cys. The Arg/N-end rule pathway involves the N-terminal arginylation (Nt-arginylation) of protein substrates and also the targeting of unacetylated destabilizing N-terminal residues (including Arg) by specific E3 N-recognins that contain the evolutionary conserved UBR domain (Fig. 1A) [16], [17], [20], [27], [28], [33], [44]–[49]. The ‘primary’ destabilizing N-terminal residues Arg, Lys, His, Leu, Phe, Tyr, Trp, and Ile are directly recognized by E3 N-recognins of the Arg/N-end rule pathway, whereas N-terminal Asp, Glu, Asn, Gln, and Cys function as destabilizing residues through their preliminary modifications. These modifications include Nt-arginylation by the Ate1 arginyl-transferase (R-transferase) (Fig. 1A) [17], [46], [47], [50], [51].

Regulated degradation of specific proteins by the Arg/N-end rule pathway mediates a legion of physiological functions, including the sensing of heme, nitric oxide (NO), oxygen and short peptides; the selective elimination of misfolded proteins; the regulation of DNA repair and cohesion/segregation of chromosomes; the signaling by G proteins; the regulation of peptide import, meiosis, apoptosis, viral and bacterial infections, fat metabolism, cell migration, actin filaments, spermatogenesis, neurogenesis, and cardiovascular development; the functioning of adult organs, including the brain, muscle, testis and pancreas; and the regulation of leaf, shoot and seed development in plants (refs. [15]–[19], [27], [32], [46], [47], [50], [51]–[53] and refs. therein).

In the yeast *Saccharomyces cerevisiae*, the Arg/N-end rule pathway is mediated by the RING-type Ubr1 E3 Ub ligase. The type-1 and type-2 substrate-binding sites of Ubr1 recognize the unmodified basic (Arg, Lys, His) and bulky hydrophobic (Leu, Phe, Tyr, Trp, Ile) N-terminal residues, respectively [16], [17], [28], [30], [33]. The type-1 binding site of Ubr1 resides in the ∼70-residue UBR domain [16], [54] that has been solved at atomic resolution [20], [48], [49]. In addition to its type-1/2 binding sites, Ubr1 contains substrate-binding sites that recognize internal (non-N-terminal) degrons of proteins that include Cup9, Mgt1, and misfolded proteins [44], [52], [55]–[60]. Recent work showed that the Ubr1-based targeting ensemble is a physical complex of the RING-type Ubr1 E3 and the HECT-type Ufd4 E3, together with their cognate E2 enzymes [26], [27], [56]. N-recognins of the mammalian Arg/N-end rule pathway comprise at least four E3 Ub ligases, UBR1, UBR2, UBR4 and UBR5, all of which contain a UBR domain [11], [17], [18], [20], [28], [34], [48], [49], [54], [61], [62]. The 200 kDa mammalian UBR1 and UBR2 are highly sequelogous (similar in sequence [63]) to each other and to the 225 kDa *S. cerevisiae* Ubr1, but are largely nonsequelogous (outside of their UBR domains) to other N-recognins such as UBR4 and UBR5.

Given the multiplicity and a partial functional redundancy of mammalian N-recognins, including the sequelogous UBR1 and UBR2 [18], [34], [64], the Arg/N-end rule pathway is still present (at a lower level of activity) in either JBS patients or *Ubr1^−/−^* mice [6], [11]. Most of the known JBS-causing changes of human *UBR1* are nonsense, frameshift or splice-site mutations that are either certain or very likely to completely abolish UBR1 activity [6]. We report here novel single-residue changes of UBR1 in patients with milder variants of JBS. These changes and one previously reported missense mutation involve amino acid residues that are conserved between the 200-kDa human UBR1 and the 225-kDa *S. cerevisiae* Ubr1 (Fig. 1B, C). Taking advantage of this evolutionary conservation, we constructed alleles of *S. cerevisiae UBR1* that were counterparts of missense *UBR1* alleles, and examined the resulting Ubr1 proteins for their activity in the *S. cerevisiae* Arg/N-end rule pathway.

## Results

### Clinical findings

**Figure 2:**
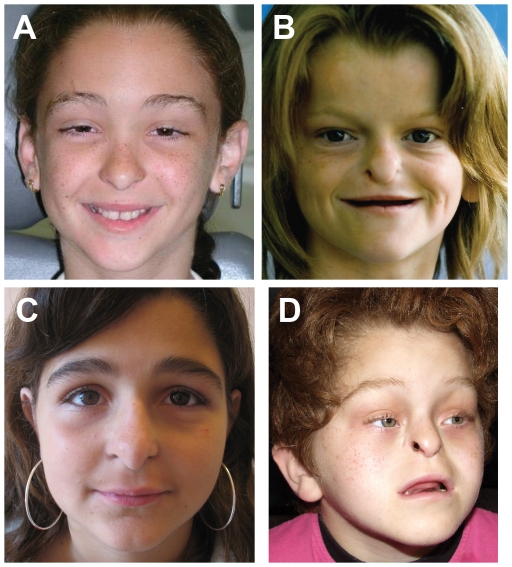
JBS patients. (A) Patient #1, whose facial appearance is nearly normal. Note the frontal upsweep of the hair and subtle hypoplasia of the nasal wings (minor signs of JBS). (B) Patient #2, a typical facial appearance of JBS, including the aplasia of nasal wings, midface hypoplasia, and a characteristic frontal hair pattern. (C) Patient #3, with a mild hypoplasia of nasal wings. (D) A previously described case of severe JBS, with typical facial features, in which both alleles of *UBR1* were, most likely, null alleles (see Results and ref. [6]).

Clinical characteristics of three patients whose *UBR1* mutations were analyzed in this study are summarized in Table 1. All patients represented sporadic cases and were born to healthy unrelated parents of European origin. Patient #1 was a 17 year old female with congenital pancreatic insufficiency and subtle facial signs of JBS (Fig. 2A). She had a small scalp defect at birth and developed mild sensorineural deafness (∼30 dB) requiring no hearing aids so far. This patient exhibited mild developmental delay and learning difficulties. She has completed a secondary school with support and is involved in a vocational training program to become ‘health assistant’. Based on her relatively high (for a JBS patient) mental status and moderate JBS-type physical and physiological anomalies, she was classified as having a mild form of JBS.

**Table 1:** Clinical features in JBS patients.

Patient	Patient #1	Patient #2	Patient #3		
Genotype	p.V122Lp.H774SfsX5	p.H136Rc.2254+2T>C	p.Q1102Ep.R503X	Total(n = 58)^a^	Biallelic nonsense/frameshift mutations (n = 12)^b^
Pancreatic insufficiency	+	+	+	49/50 (98%)	12/12 (100%)
Dental defects	+	+	+	27/28 (96%)	6/6 (100%)
Nasal wing hypoplasia	very mild	+	mild	54/55 (98%)	12/12 (100%)
Deafness	mild	+	+	31/45 (69%)	10/11 (91%)
Scalp defect	+	+	+	41/57 (72%)	8/12 (67%)
Hypothyroidism	-	+	-	17/46 (37%)	5/10 (50%)
Short stature	-	+	-	32/38 (84%)	8/9 (89%)
Urogenital anomalies	-	+	-	15/51 (29%)	5/12 (42%)
Imperforate anus	-	+	-	20/58 (34%)	6/12 (50%)
Mental retardation	-	+	-	25/36 (69%)	8/8 (100%)

a Ref. 1;

b Ref. 6 and unpublished data.

Patient #2 was a 14 year old female with a typical clinical picture of severe JBS (Fig. 2B; *cf*. Fig. 2D). Her genotype has been reported previously [6] (Fig. 1B). In addition to the typical nasal wing aplasia and congenital pancreatic insufficiency, patient #2 also exhibited scalp defects, anal atresia, renal anomalies, hypothyroidism, severe deafness, oligodontia, and short stature. Her cognitive performance was in the mentally retarded range (IQ 50–60).

Patient #3 was a 10 year old girl who was diagnosed with mild JBS, based on the presence of pancreatic insufficiency and mild facial anomalies (Fig. 2C). She was born with a small scalp defect at the vertex and has been wearing hearing aids since she was 4 years old. Many permanent teeth are missing. The girl is attending a special school for children with hearing impairments. Her cognitive level is reported to be in the low normal range. No formal IQ testing has been done so far.

### *UBR1* mutations in JBS patients

Patients #1–3 were compound *UBR1* heterozygotes. Specifically, each of them carried a missense mutation in one *UBR1* allele and a mutation in the other *UBR1* allele that would be, most likely, a null mutation. Patient #1 was compound heterozygous for the missense mutation c.364G>C (p.V122L) in exon 3 (Fig. 1B) and a 1 bp duplication (c.2319dupT) in exon 21. The latter *UBR1* mutation resulted in a translational frameshift and a premature stop codon (p.H774SfsX5). Patient #2 was compound heterozygous for the exon 3 missense mutation c.407A>G (p.H136R) in exon 3 (Fig. 1B) and a mutation at the splice donor site of exon 20 (c.2254+2T>C). The latter mutation is predicted to cause a skipping of the 64-bp exon 20, resulting in a shift of the *UBR1* open reading frame (ORF) and premature stop codon (Mutation Taster: www.mutationtaster.org/). Patient #3 carried a missense mutation c.3304C>G (p.Q1102E) in exon 30 of one *UBR1* allele (Fig. 1C) and a nonsense mutation c.1507C>T (p.R503X) in exon 13 of the other allele.

A preferential expression of the corresponding missense *UBR1* alleles was observed with patients #1 and #3, whose blood leukocyte RNA samples were available (data not shown), consistent with the (presumed) nonsense-mediated decay (NMD) of mutant *UBR1* mRNAs that were transcribed from the *UBR1* alleles containing the frameshift and nonsense mutations in patients #1 and #3, respectively. Among the three missense alleles of *UBR1*, two of them, V122L (patient #1) and Q1102E (patient #3), are novel (Fig. 1B, C). The H136R mutation in *UBR1* of patient #2 was described by us previously [6]. None of these *UBR1* mutations were found in more than 300 healthy control subjects. The three affected positions in UBR1 proteins of patients #1–3 are sufficiently highly conserved to have unambiguously identifiable counterparts in *S. cerevisiae* Ubr1 and in other eukaryotes as well (Fig. 1B, C).

**Figure 3:**
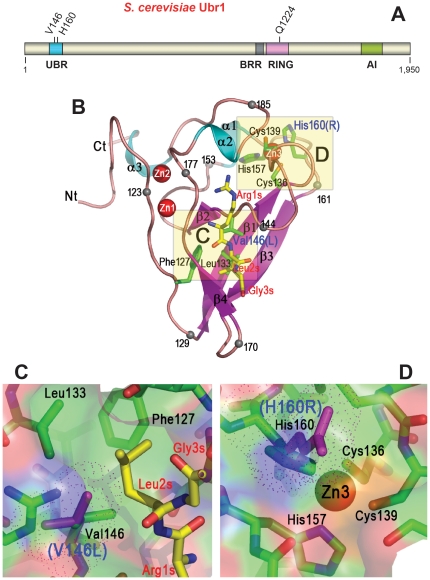
*S. cerevisiae* Ubr1 N-recognin. (A) A diagram of the 225 kDa *S. cerevisiae* Ubr1. The indicated evolutionarily conserved regions of Ubr1 are the UBR box, the BRR (basic residues-rich) domain, the Cys/His-rich RING-H2 domain, and the AI (autoinhibitory) domain [18], [30], [33]. Three missense mutations in patients #1-3 of the present work are indicated as well (see Fig. 1B, C). (B) Ribbon diagram of the *S. cerevisiae* UBR domain [48] in a complex with RLGES, the N-terminal region of the separase-produced fragment of Scc1, a subunit of cohesin [75]. The bound RLGES peptide is shown as a stick model, with carbon atoms colored yellow. Several residues are marked with a black sphere and numbered to facilitate the tracing of the polypeptide chain. The names of residues of the RLGES peptide are in red, with the letter ‘s’ (substrate) appended to their position numbers. Side-chains of residues in the UBR domain that are present near JBS mutations (Fig. 1B, C) are shown in a stick form, with carbon atoms colored green. Three coordinated zinc ions of the UBR domain [48] are shown as red spheres. (C) Close-up view of the UBR region near the V146L mutation (patient #1; Fig. 1B). In panel B, this region of UBR is boxed and labeled as ‘C’. The residues of UBR that accommodate the position-2 Leu residue (‘Leu2s’) of the RLGES peptide substrate are shown and labeled. The van der Waals sphere of the mutant Leu residue, in the UBR1^V146L^ mutant, is shown as purple dots. (D) Close-up view of the UBR region near the H160R mutation (patient #2, Fig. 1B). In panel B, this region of UBR is boxed and labeled as ‘D’. The residues of UBR coordinating Zn3 atom are shown and labeled. The van der Waals sphere of the mutant Arg residue, in the UBR1^H160R^ mutant, is shown as purple dots. The views in (C) and (D) are oriented to maximize visibility of mutation-proximal residues.

The V122L mutation in patient #1 and the H136R mutation in patient #2 are located in the N-terminus-proximal UBR box of human UBR1 (and *S. cerevisiae* Ubr1), whereas the Q1102E mutation affects the RING-H2 domain in the C-terminal half of Ubr1 (Figs. 1B, C and 3A). Positions 122, 136 and 1,102 of the human UBR1 protein correspond to positions 146, 160, and 1,224 of *S. cerevisiae* Ubr1 (Fig. 1B, C). Fig. 3A, B illustrates the overall organization of the 225 kDa *S. cerevisiae* Ubr1 and the structure of its UBR domain [48]. This crystal-derived structure of UBR in yeast Ubr1 is highly spalogous (spatially similar [63]) to the crystal structure of the human UBR domain [20], [49]. Fig. 3B–D illustrates, through molecular modeling, the spatial configurations of locales in the structure of the UBR domain that contain single-residue JBS alterations. These models were produced by mutating specific residues of wild-type UBR1 *in silico* and thereafter choosing rotamers of these residues to minimize steric clashes (Fig. 3C, D).

The wild-type Val^146^ residue of yeast Ubr1 (Val^122^ in human UBR1) is located immediately before a short β-strand that forms in the UBR domain upon its binding to a peptide with N-terminal Arg (a mimic of N-end rule substrate) (Fig. 3B). This region of the UBR domain exists as a loop in the absence of a bound peptide [48]. Because the side chain of Leu is larger than that of Val, the V146L alteration of Ubr1 (Fig. 1B) is expected to locally perturb UBR conformation, but not in a major way. In Fig. 3C, the second residue of the UBR-bound peptide is Leu, denoted as ‘Leu2s’, i.e., Leu^2^ of substrate. One testable possibility is that the V146L mutation decreases the affinity of the UBR domain for type-1 N-end rule substrates with position-2 residues that are bulkier than Leu.

As to the H160R mutation, i.e. the other missense JBS alteration in the UBR domain, (H136R in human UBR1), its likely functional consequences are more clear and more severe, because wild-type His^160^ is one of two histidines and two cysteines that coordinate Zn3, a third zinc ion in the UBR domain (Fig. 3B, D). A bulky and strongly positively charged residue such as Arg at this position is likely to destabilize coordination of Zn3 (Fig. 3D). In contrast to the wild-type Ubr1, Ubr1^V146L^ and Ubr1^Q1224E^ proteins, Ubr1^H160R^ was expressed at low steady-state levels both in *S. cerevisiae* and in lymphocytes of patient #2, strongly suggesting its metabolic instability (see below). Finally, although a 3-D structure of the RING-H2 domain, in the C-terminal half of Ubr1 (Fig. 3A), is unknown, it is likely that the replacement of the highly conserved uncharged Gln^1224^ (Gln^1102^ in human UBR1) by the charged Glu residue in Ubr1^Q1224E^ (Fig. 1C) would significantly perturb RING-H2 (a Zn-stabilized domain in RING-type E3 Ub ligases [24], [65], [66]. The function of the RING-H2 domain in Ubr1 includes the interaction of this E3 with a cognate E2 enzyme (Rad6 in *S. cerevisiae*, HR6A or HR6B in mammals) [11], [17], [34], [67], [68].

### Functional testing of *S. cerevisiae* JBS-type Ubr1 mutants

Low-copy (*CEN*-based) plasmids that expressed the wild-type *S. cerevisiae* Ubr1 and its single-residue mutants Ubr1^V146L^, Ubr1^H160R^ and Ubr1^Q1224E^ (Fig. 1B, C) from the native yeast P*_UBR1_* promoter, were transformed into *ubr1Δ* cells that lacked Ubr1 and therefore lacked the Arg/N-end rule pathway. These cells also carried plasmids that expressed the previously characterized X-β-galactosidase (X-βgal) N-end rule reporters, produced using the Ub fusion technique, i.e. through the cotranslational deubiquitylation, by a family of deubiquitylase enzymes, of Ub-X-βgal fusion proteins (X = His, Tyr) [27], [30]–[32], [69]. The His and Tyr residues of His-βgal and Tyr-βgal are examples of the type-1 and type-2 primary destabilizing N-terminal residues (Fig. 1A). These residues are recognized by the corresponding binding sites of Ubr1 (see Introduction). As shown previously, the enzymatic activity of βgal in extracts from yeast cells that express an X-βgal reporter can serve as a reliable measure of the reporter's metabolic stability [30], [31], [69]. We chose His and Tyr as the N-terminal residues of X-βgal reporters for these assays, instead of, for example, the more ‘destabilizing’ type-1 and type-2 N-terminal residues such as Arg or Leu. The moderately destabilizing His (type-1) and Tyr (type-2) residues resulted in a slower degradation of the corresponding N-end rule reporters in wild-type cells, thereby increasing the sensitivity of this assay to changes in Ubr1 activity.

**Figure 4:**
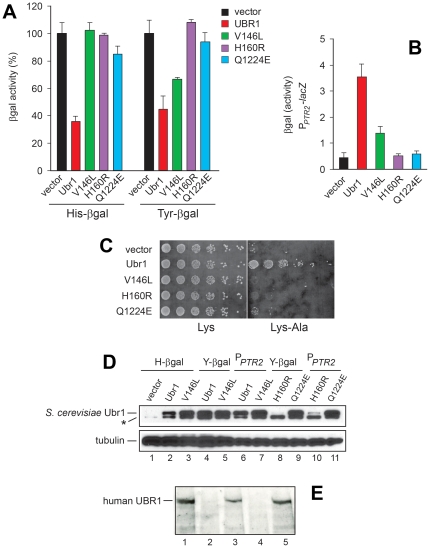
Functional activity of yeast Ubr1 mimics of missense JBS-UBR1 mutants. (A) Relative enzymatic activity of βgal in extracts from *S. cerevisiae* JD55 (*ubr1Δ*) that expressed His-βgal or Tyr-βgal, and also carried an empty vector, or an otherwise identical plasmid expressing wild-type *S. cerevisiae* Ubr1, or (separately) its three missense mutants Ubr1^V146L^, Ubr1^H160R^, or Ubr1^ Q1224E^. The activity of βgal was measured in triplicates, with standard deviations shown. (B) Relative levels of induction of the peptide transporter Ptr2 were assayed by measuring the activity of a plasmid-borne *lacZ* (βgal-encoding) reporter that was expressed from the P*_PTR2_* promoter in *ubr1Δ S. cerevisiae* that carried either an empty vector or otherwise identical plasmids that expressed either wild-type Ubr1 [27], [28], [52] or its indicated mutants. Cells were grown to A_600_ of ∼0.8 in SC(-Ura, -Leu) medium at 30°C, followed by measurements, in triplicate, of βgal activity in cell extracts, with standard deviations shown. (C) The lysine-requiring JD55 (*ubr1Δ*) *S. cerevisiae* strain was grown on plates containing 110 µM lysine (Lys) or 66 µM Lys-Ala dipeptide as the sole source of Lys in the medium [27], [33], [52]. JD52 (*ubr1Δ*) cells carried a vector plasmid or otherwise identical plasmids expressing wild-type Ubr1 or its missense mutants Ubr1^H160R^, Ubr1^V146L^ and Ubr1^ Q1224E^. Cells were grown to A_600_ of ∼1 in SC(-Leu) medium at 30°C, washed in sterile water, serially diluted 5-fold, spotted on SC(-Leu, -Lys) plates containing 110 µM Lys or 66 µM Lys-Ala, and incubated at 30°C for 3 days. (D) Cell extracts (equal total protein levels) from experiments described in panels A and B were subjected to SDS-PAGE, followed by immunoblotting with affinity-purified anti-Ubr1 antibody (upper panel) and anti-tubulin antibody (a loading control; lower panel). Asterisk indicates a protein that crossreacts with anti-Ubr1 antibody. (E) Extracts from human lymphocytes (equal amounts of total protein) were subjected to SDS-PAGE, followed by immunoblotting with antibody to human UBR1 (see Materials and Methods). Lane 1, wild-type lymphocytes. Lane 2, same as lane 1 but from lymphocytes of patient #2 (see the main text and Figs. 1 and 2). Lane 3, same as lane 1 but with lymphocytes from patient #3. Lane 4, same as lane 1, but with lymphocytes from a JBS patient with a homozygous nonsense mutation in *UBR1*, previously shown to have no detectable UBR1 (null UBR1 control) [17]. Lane 5, same as a lane 1.

Steady-state levels of His-βgal and Tyr-βgal were significantly decreased in cells that expressed wild-type Ubr1, in comparison to their levels in *ubr1Δ* cells, owing to degradation of these reporters by the Arg/N-end rule pathway [27], [30], [31], [69] (Fig. 4A). Ubr1^H160R^, whose single-residue mutation resides in the UBR domain, in the region of the Zn3 ion coordination that is expected to be strongly perturbed by the change from His to Arg at position 160 (Figs. 1B, 3D, and discussion above), was completely inactive in conferring metabolic instability on His-βgal or Tyr-βgal (Fig. 4A). The absence of detectable activity in Ubr1^H160R^ resulted, most likely, from the above structural perturbation but could be also caused, in part, by the metabolic instability of Ubr1^H160R^ (see below). The same measurements with Ubr1^Q1224E^, whose single-residue mutation resides in the RING-H2 domain (Figs. 1B, C and 3A) indicated a much lower than wide-type but reproducibly detectable activity of Ubr1^Q1224E^ toward both His-βgal and Tyr-βgal (Fig. 4A). Specifically, the levels of these reporters in the presence of Ubr1^Q1224E^ were slightly but reproducibly lower than the levels of the same reporters in *ubr1Δ* cells that carried empty vector (Fig. 4A).

Ubr1^V146L^, a mimic of the missense JBS mutation in UBR1 of patient #1 (Figs. 1B, 2A, and 3C), was apparently inactive in conferring metabolic instability on His-βgal but exhibited a reproducibly significant activity with Tyr-βgal (Fig. 4A). Ubr1 recognizes N-terminal His, a type-1 destabilizing residue, via its type-1 binding site, which resides in the UBR domain, i.e., the region of mutation in Ubr1^V146L^ (Fig. 1B and Fig. 3B, C). As discussed above, the severity of perturbation of the UBR domain by this mutation (V146L) is predicted to be lower than the one by H160R. Thus, a parsimonious interpretation of these results is that a functional perturbation of the UBR domain in Ubr1^V146L^ abolishes (or nearly abolishes) its activity toward type-1 N-end rule substrates but only impairs (does not abolish) its targeting of type-2 N-end rule substrates, exemplified by Tyr-βgal (Fig. 4A). The N-terminal Tyr residue is recognized by the type-2 site of Ubr1, located downstream of the UBR domain [16], [18], [19], [28]. In sum, a decreased but significant activity of Ubr1^V146L^ in targeting Tyr-βgal (Fig. 4A) is consistent with a lower extent of (expected) perturbation of the UBR domain by this mutation, in comparison to the one in Ubr1^H160R^ (Fig. 3B–D).

Remarkably, the absence of detectable functional activity in yeast Ubr1^H160R^ (the mimic of human UBR1 in patient #2), versus the presence of residual activities in both yeast Ubr1^V146L^ and Ubr1 ^Q1224E^ (the mimics of human UBR1 in patients #1 and #3, respectively) (Fig. 4A), correlated with a stronger clinical expression of JBS symptoms in patient #2, in comparison to patients #1 and #3 (Table 1, Fig. 2, and discussion above).

### Regulation of peptide import by wild-type and mutant Ubr1 proteins

The binding of short peptides with destabilizing N-terminal residues to the type-1/2 sites of Ubr1 (see Introduction) allosterically activates the autoinhibited third substrate-binding site of Ubr1 that recognizes an internal degron of Cup9, a transcriptional repressor of roughly 50 genes [17], [33], [44], [52], [55]. Genes that are down-regulated by Cup9 include *PTR2*, which encodes the transporter of di- and tripeptides [70]. The resulting Ubr1-Cup9-Ptr2 positive-feedback circuit, in which the Ubr1-mediated degradation of the Cup9 repressor is accelerated by type-1/2 peptides that bind to Ubr1, allows *S. cerevisiae* to sense the presence of extracellular peptides and to react by accelerating their uptake through induction of the Ptr2 transporter [44], [52], [55]. A previously characterized cell growth assay allows comparisons of the efficacies of dipeptide import by congenic *S. cerevisiae* strains [27], [33], [52]. In this assay, a lysine-requiring *S. cerevisiae* strain is grown on plates containing either lysine (Lys) or the Lys-Ala dipeptide as the sole source of Lys in the medium. To grow under the latter conditions, cells must be capable of a sufficiently efficacious dipeptide import. *ubr1Δ S. cerevisiae* carrying either a vector plasmid or otherwise identical plasmids expressing wild-type Ubr1 or its missense mutants Ubr1^H160R^, Ubr1^V146L^ and Ubr1 ^Q1224E^, were grown in the presence of either Lys or Lys-Ala in the medium (Fig. 4C). Whereas all examined strains grew in the presence of Lys, only cells expressing wild-type Ubr1 grew on plates containing Lys-Ala instead of Lys (Fig. 4C).

In a different assay for peptide import, relative levels of induction of the peptide transporter Ptr2 were assayed by measuring the activity of a *lacZ* (βgal-encoding) reporter that was expressed from the P*_PTR2_* promoter in *ubr1Δ S. cerevisiae* that carried either an empty vector or otherwise identical plasmids that expressed wild-type Ubr1 [27], [28], [52] or its missense mutants. In contrast to wild-type Ubr1, which strongly induced the P*_PTR2_-lacZ* fusion, both Ubr1^H160R^ and Ubr1^Q1224E^ mutants did not induce it detectably, i.e., significantly above the level in the presence of vector alone (Fig. 4B). Interestingly, the Ubr1^V146L^ mutant, similarly to its reduced but still significant activity in mediating the *in vivo* degradation of Tyr-βgal (Fig. 4A), exhibited a diminished but significant activity in the P*_PTR2_*-*lacZ* assay (Fig. 4B).

As a part of Ubr1 tests, we also compared the levels of wild-type and mutant Ubr1 proteins that were produced from the native P*_UBR1_* promoter and low copy plasmids in *ubr1Δ S. cerevisiae* (see Materials and Methods). Cell extracts from indicated *S. cerevisiae* transformants were subjected to SDS-PAGE and immunoblotting with the previously characterized, affinity-purified antibody to yeast Ubr1 [33]. Similar amounts of Ubr1 and its mutants were produced in yeast transformants that had been employed in experiments of this study, except for Ubr1^H160R^, whose levels were considerably lower than the levels of either wild-type Ubr1, Ubr1^Q1224E^ or Ubr1^V146L^ (see Fig. 4D and its legend for details). The Ubr1-expressing plasmids were identical save for single-nucleotide nonsynonymous mutations in the *UBR1* ORF (Fig. 1B, C). Thus a parsimonious interpretation is that the H160R mutation, which is expected to strongly destabilize the UBR domain (Fig. 3D) (see discussion above), results, in turn, in a metabolic destabilization and low steady-state levels of the Ubr1^H160R^ protein (Fig. 4D).

This interpretation is strongly supported by independent evidence, through immunoblotting-based comparisons of the levels of human UBR1 proteins in lymphocytes of JBS patients (Fig. 4E). Whereas the mutant UBR1 ^Q1102E^ protein of patient #3 was readily detectable in lymphocytes of this patient, no UBR1 could be detected in otherwise identical extracts from patient #2, whose UBR1 ^H136R^ was the counterpart of yeast UBR1 ^H160R^ (Fig. 4E). We conclude that the absence of detectable Ubr1^H160R^ activity *in vivo*, in contrast to Ubr1^Q1224E^ and Ubr1^V146L^ (Fig. 4A, B), stemmed, at least in part, from the accelerated *in vivo* degradation of Ubr1^H160R^, in addition to the likely diminished or absent functional activity of this mutant. A precedent for a single missense mutation that could confer a short *in vivo* half-life on yeast Ubr1 was the previously characterized change of its wild-type Tyr^277^ to Ala or Glu [33].

## Discussion

Mutational inactivation of human UBR1, one of the E3 Ub ligases of the Arg/N-end rule pathway (Fig. 1A), is the cause of Johanson-Blizzard syndrome (see Introduction) [1], [2], [6], [17]. Previously studied cases of the typical severe expression of the syndrome involved nonsense, frameshift or splice-site mutations of *UBR1* that were either certain or very likely to completely abolish UBR1 activity [6]. The present study of less severe JBS cases and their association with missense mutations in one of two copies of *UBR1* indicates that the relative mildness of symptoms in JBS patients #1 and #3 (Fig. 2A, C) is most likely caused by a significant residual activity of the corresponding UBR1 mutants (Figs. 1B, C and 4A–C).

The mechanistic cause(s) of JBS remains to be understood, in part because all other UBR-type N-recognins, including UBR2 (which is 47% identical to UBR1 [11], [34] and is expressed in exocrine pancreas as well) are retained in JBS patients. Their cells, therefore, still contain the Arg/N-end rule pathway. One possibility is that UBR1, despite its strong sequelogy [63] to UBR2, has a physiological protein substrate(s) that is unique to UBR1. If so, a loss of UBR1 activity (for example, its total loss in severe JBS (Fig. 2D) [6]) would increase the level of a postulated (normally short-lived) substrate(s) and thereby mediate (or contribute to) the broad range of JBS phenotypes, with severity of these phenotypes determined by the levels of residual UBR1 activity in specific cell types of a JBS patient. Alternatively, physiological substrates that are not unique to UBR1 might be involved. Previous work has shown that *S. cerevisiae* Ubr1 is an activity-limiting component of the yeast Arg/N-end rule pathway [71]. Thus UBR1 and UBR2 may share all of JBS-relevant physiological substrates but in the absence of UBR1 the efficacy of targeting of such substrates by UBR2 alone might not be high enough, particularly in some cell types. (Expression patterns of mouse *Ubr2* overlap with but are not identical to those of *Ubr1* [11], [34].)

It is also possible that a JBS-relevant function of UBR1 is a previously unknown and *a priori* unexpected one. For example, it was recently shown that mouse Ubr2, a strong sequelog of Ubr1 (47% identity in mice), functions to metabolically *stabilize* Tex19.1, a germ cell-specific protein in mouse testis, through a direct interaction between Ubr2 and Tex19.1 [62]. Metabolic stabilization of Tex19.1 by Ubr2 in wild-type mouse cells is functionally relevant, because both *Tex19.1^−/−^* mice and *Ubr2^−/−^* mice exhibit similar phenotypes of defective spermatogenesis, and the levels of Tex.19.1 in testis are strongly decreased in the absence of Ubr2 [62]. It is unknown, at present, whether Ubr1 also binds to and stabilizes Tex19.1. However, it is already clear that at least some N-recognins not only target proteins for degradation but can also bind to and protect specific proteins from degradation *in vivo* [17], [62], a circumstance that further increases the range of UBR1 mechanisms that may be relevant to JBS.

A major lacuna in the current understanding of mammalian N-recognins is the paucity of identified physiological UBR1 substrates. At present, the known (as distinguished from putative) substrates of mammalian UBR1 comprise largely the G-protein regulators RGS4, RGS5 and RGS16, and the separase-produced fragment of the Rad21 cohesin subunit (refs. [16], [17], [51], [72] and refs. therein). Misfolded proteins are also among physiological substrates of UBR1 and UBR2 in mammals and Ubr1 in yeast, although specific degrons involved remain to be identified [57], [58], [59], [60]. In addition, physiological substrates of *S. cerevisiae* Ubr1 include Cup9 and Mgt1, a transcriptional repressor and a DNA repair protein, respectively (see Introduction). For several reasons [16], [17], it is highly likely that mammalian UBR1 and other eukaryotic N-recognins have a large number of physiological substrates. Identifying such proteins (Fig. 1A), with an emphasis on substrates that might be unique for UBR1 (as distinguished, for example, from UBR2), should advance the mechanistic understanding of JBS and its multiple phenotypes.

## Materials and Methods

### Patients

This study was approved by the Local Ethics Committee (University Hospital, Magdeburg, Germany), and informed consent, in writing, was obtained from the parents/patients, including written informed consent for publication of the present data in biomedical journals, including PLoS One. Patients were personally evaluated by a clinical geneticist (M.C.A, A.P.A, H.B.) and their hospital charts were reviewed. These patients are a part of the cohort of 35 unrelated, molecularly confirmed JBS patients that were identified over several years. The criterion for inclusion in this study was the presence of a missense *UBR1* mutation affecting an amino acid residue at a position conserved between human UBR1 and *S. cerevisiae* Ubr1.

### Mutations in *UBR1*

Genomic DNA was extracted from peripheral blood leukocytes using standard methods. All 47 coding exons of the human *UBR1* gene and flanking intronic regions were amplified by PCR and subjected to bidirectional sequencing using the dye-terminator sequencing method (BigDye Terminator v.3.1; Applied Biosystems) and an automated capillary sequencer ABI 3730 Genetic Analyzer, (Applied Biosystems, Weiterstadt, Germany), as described previously [6].

### Yeast strains, plasmids, β-galactosidase assays, and immunoblotting

The *S. cerevisiae* strains used were JD52 (MATa *ura3-52 his3-Δ200 leu2-3,112 trp1-Δ63 lys2-801 ubr1Δ::HIS3*) and JD55 (MATa *ura3-52 his3-Δ200 leu2-3,112 trp1-Δ63 lys2-801 ubr1Δ::HIS3*) [56]. The low-copy plasmids were the previously described pRS315 (control vector) and pCH100 (pRS315-UBR1) [32], [33], or the otherwise identical pCH638 (pRS315-UBR1^H160R^), pCH639 (pRS315-UBR1^Q1224E^), and pCH640 (pRS315-UBR1^V146L^) that expressed Ubr1 mutants. The pCH100 plasmid contained only one of the *Stu*I, *Spe*I, *Msc*I and *Mlu*I sites in *S. cerevisiae UBR1*. Overlapping-extension PCR was used to introduce specific mutations (V146L, H160R and Q1224E) into the *UBR1* ORF. A pair of PCR primers, OOM7/OOM8 or OCH56/OCH88 (Table 1), which flanked the region between the *Stu*I and *Spe*I sites, or between the *Msc*I and *Mlu*I sites of *UBR1*, were used to construct V146L, H160R and Q1224E UBR1 mutants. To do so, pCH100 was employed as a PCR template, in conjunction with specific primers (Table 1). The resulting PCR products were digested with *Stu*I/*Spe*I or *Msc*I/*Mlu*I and ligated into *Stu*I/*Spe*I-cut or *Msc*I/*Mlu*I-cut pCH100, yielding the plasmids pCH638, pCH640 and pCH639, respectively.

Standard yeast techniques and media were employed for strain construction, transformation and growth [73], [74]. Assays for β-galactosidase (βgal) activity in *S. cerevisiae* extracts were carried out as previously described [27], [30], [31], [69], using Yeast β-Galactosidase Assay Kit (Thermo scientific, Rockford, IL) and the manufacturer's protocol. Immunoblotting of proteins that had been fractionated by SDS-4–12% NuPAGE was carried out as previously described, using a previously characterized, affinity-purified antibody to *S. cerevisiae* Ubr1 [27], [32], [56]. Immunoblotting of extracts from human lymphocytes was carried out using antibody to human UBR1, as previously described [6].

JD55 (*ubr1Δ*) *S. cerevisiae* carried the plasmids pSS4 (P*_PTR2_-LacZ*) and either pCH100 (wild-type Ubr1), pCH638 (Ubr1^H160R^), pCH639 (Ubr1^Q1224E^), or pCH640 (Ubr1^V146L^). Cells were grown at 30°C in synthetic complete (SC) medium (0.17% yeast nitrogen base, 0.5% ammonium sulfate, 2% glucose, plus a dropout mixture of compounds required by a given auxotrophic strain) to A_600_ ∼0.8, followed by the measurements of βgal activity in cell extracts.
